# Spinal cord involvement in Kearns-Sayre syndrome: a neuroimaging study

**DOI:** 10.1007/s00234-020-02501-0

**Published:** 2020-07-22

**Authors:** Pasquini Luca, Guarnera Alessia, Rossi-Espagnet Maria Camilla, Napolitano Antonio, Martinelli Diego, Deodato Federica, Diodato Daria, Carrozzo Rosalba, Dionisi-Vici Carlo, Longo Daniela

**Affiliations:** 1 Neuroradiology Unit, Imaging Department, Bambino Gesù Children’s Hospital, P.zza Sant’Onofrio 4, 00165 Rome, Italy; 2 Neuroradiology Unit, NESMOS Department, Sant’Andrea Hospital, La Sapienza University, Rome, Italy; 3 Medical Physics Department, Bambino Gesù Children’s Hospital, IRCCS, Rome, Italy; 4 Division of Metabolism, IRCCS Bambino Gesù Children’s Hospital, Rome, Italy; 5 Unit of Muscular and Neurodegenerative Disorders, Laboratory of Molecular Medicine, IRCCS Bambino Gesù Children’s Hospital, 00146 Rome, Italy

**Keywords:** Kearns-Sayre syndrome, Spinal cord, MRI, Mitochondrial disease

## Abstract

**Purpose:**

Spinal cord involvement in Kearns-Sayre (KSS) syndrome could be more frequent than commonly thought. Our aims were to evaluate the involvement of the spinal cord in patients with KSS by means of MRI and to investigate possible correlations of spinal and brain disease with patient disability.

**Methods:**

Eleven patients with KSS disease and spinal cord MRI were retrospectively recruited. The severity of spinal disease was defined as follows: grade 0 (none), grade 1 (focal), and grade 2 (extensive). We calculated a radiologic score of brain involvement based on typical features. We performed a chi-square test to correlate spinal cord and brain MRI involvement to patient disability. For significant variables, a contingency coefficient, phi factor, and Cramer’s *V* were also computed.

**Results:**

Spinal cord lesions were detected in 6/11 patients, showing four patterns: involvement of gray matter, gray matter and posterior columns, posterior columns, and anterior columns. The severity of spinal disease was grade 1 in two and grade 2 in four patients. All patients showed brain involvement (9-point average for patients with spinal involvement and 10 for the others). A significant correlation was found between disability score and spinal cord involvement (*χ*^2^ = 7.64; *p* = 0.022) or brain score (*χ*^2^ = 26.85; *p* = 0.043). Significance for brain score-disability correlation increased with the spinal cord as a cofactor (*χ*^2^ = 24.51; *p* = 0.017, phi factor = 1.201, Cramer’s *V* = 0.849, contingency effect = 0.767; *p* = 0.017).

**Conclusion:**

Spinal cord lesions are common in KSS. Patients with spinal disease show higher disability than patients without spinal cord lesions, supporting the inclusion of dedicated acquisitions to routine MRI of the brain in patients with KSS.

## Introduction

The Kearns-Sayre syndrome (KSS) is a rare mitochondrial encephalopathy, with an estimated prevalence of around 1/125,000 [1]. The syndrome is characterized by onset before 20 years of age and a typical clinical triad: pigmentary retinopathy (PR), progressive external ophthalmoplegia (PEO), and cardiac conduction anomalies including heart block [1, 2]. Clinical manifestations may also include cerebellar ataxia, muscle weakness, growth failure, dementia, bilateral sensorineural hearing loss, endocrinopathies including diabetes, cerebrospinal fluid (CSF) protein concentration exceeding 100 mg/dL (>1 g/L), elevated lactate, and pyruvate in blood and CSF [1].

The syndrome most commonly associates with a single large-scale mitochondrial DNA (mtDNA) deletion, more rarely to point mutations, often sharing most of the phenotype with the Pearson syndrome [1].

The most common brain magnetic resonance imaging (MRI) findings in KSS are T2/FLAIR hyperintense, bilateral, and symmetric lesions observed in cerebral and cerebellar white matter (WM), thalamus, basal ganglia, and brainstem. U-fibers are predominantly involved while periventricular WM is usually spared. Cerebral and cerebellar atrophy is also a frequent finding [3].

Despite multiple reports of brain involvement on MRI in the literature, spinal cord disease is poorly described on imaging, and spine MRI evaluation is seldom performed in the clinical routine of patients affected by KSS [4]. Kearns and Sayre described cervical spinal cord involvement in their first post-mortem autoptic report of the disease, attributing the finding to possible post-mortem artifacts [5]. Later, autoptic reports confirmed this evidence, supporting the idea of spinal cord lesions being more common than previously thought. In fact, other mitochondrial leukodystrophies show variable spinal cord involvement, supporting the possibility of similar findings for KSS [6].

Our aim is to evaluate the involvement of the spinal cord in patients with KSS by means of MRI. Also, we investigated the possible correlation of spinal lesions and brain disease, quantified by a neuroimaging score based on typical disease locations [5, 7–9], with patient disability (meaning loss of autonomous ambulation).

## Materials and methods

### Study population

The design of the study was retrospective and observational. Informed consent was obtained from the patient/next of kin before the execution of the MRI. Our institutional review board approved the present study with a waiver of informed consent, due to retrospective observational design (Ospedale Pediatrico Bambino Gesù; protocol number 1867/2019). The study was conducted in accordance with the Declaration of Helsinki.

Patients were retrospectively recruited by reviewing the imaging archive of our institution from 2011 to 2020 with the following inclusion criteria: (a) presence of MRI exam including spinal cord examination with axial and/or sagittal T2 images; (b) genetic confirmation of KSS disease obtained with mitochondrial DNA analysis from muscle biopsy, blood, or urine samples. The only exclusion criterion was the presence of significant image artifacts.

### Imaging protocols

Patients underwent multiple MRI to assess the central nervous system (CNS) disease progression according to clinical indications. MRI was performed for each patient on the same 3 T magnet (Siemens Skyra, Siemens Medical Systems, Erlangen, Germany), with a protocol including at least axial T2-weighted (TR = 8600, TE = 122, slice thickness = 3 mm); axial FLAIR (TR = 9000, TE = 85, slice thickness = 3 mm); axial T1-weighted (TR = 500, TE = 9.9, slice thickness = 3 mm); DWI (TR = 9000, TE = 98, slice thickness = 3 mm, *b* value = 0/1000); axial and sagittal T2-weighted images for the spinal cord, including cervical, dorsal, and lumbar segments (TR = 4000, TE = 122, slice thickness = 2.3 mm).

### Imaging evaluation

Spinal cord involvement was evaluated on sagittal and axial T2-weighted images. For statistical correlation, spinal involvement was defined as a binary variable (0/1). The severity of spinal cord disease was classified in three different grades: no involvement (grade 0), focal involvement (limited to one spinal segment, grade 1), or extensive involvement (two or more spinal segments, grade 2) (Table 1). The pattern of involvement was qualitatively evaluated on axial T2-weighted images.

**Table 1:** Demographic, clinical, and neuroradiologic data in Kearns-Sayre patients at every MR timepoint

Subject	Sex	FU interval (m)	Age (y)	Disease Duration (y)	Mutation (Bp)	Brain MR Score	Spinal cord involvement	Spinal cord score	Disability
1*	F		12	9	4977	11	1	2	1
1		21	14	11		12	1	2	2
2	M		12	4	4977	4	1	2	0
2		3	12	4		4	1	2	0
2		2	12	4		4	1	2	0
2		31	15	7		9	1	2	0
3	F		19	12	6990	10	1	2	2
3		1	19	12		N/A	1	2	1
3		2	19	12		11	1	2	1
3		21	21	14		11	1	1	1
3		4	21	14		11	1	1	1
3		3	22	15		11	1	2	1
4	F		13	1	4977	11	1	1	0
5*	F		6	0	4977	8	1	2	0
5		58	11	5		14	1	2	1
6	M		20	11	7436	9	1	1	1
6		16	22	12		9	1	1	1
6		8	22	13		9	1	1	1
7	M		11	3	4977	12	0	0	0
7		35	14	6		15	0	0	0
8	F		11	6	5000	9	0	0	0
9	F		40	15	7664	14	0	0	0
10	M		15	1	5000	8	0	0	0
10		15	16	2		8	0	0	0
11	M		4	4	6720	7	0	0	0

*N/A*, missing value (the patient performed only spinal imaging at the follow-up timepoint)

*Patients deceased at the time of the study

*Bp*, base pairs; *y*, years; *m*, months

To compare spine and brain disease, we calculated a radiologic score on axial T2 images, based on typical disease locations described in previous works [5, 7–9]. The score had a maximum of 16 points, distributed as follows: subcortical white matter (1 point), deep white matter (1 point), basal ganglia (2 points for the involvement of > 2 among putamen, globus pallidus, and caudate; 1 point if less), thalamus (1 point), hypothalamus (1 point), mesencephalon (2 points for plate and tectum involvement; 1 point if less), pons (2 points for plate and tectum involvement; 1 point if less), medulla (2 points for plate and tectum involvement; 1 point if less), cerebellar white matter (1 point), cerebral atrophy (1 point), and cerebellar atrophy (1 point) (Table 1).

Images were evaluated separately by two research fellows in pediatric neuroradiology blinded from clinical data and subsequently a final consensus was obtained under the supervision of a 20-year experienced pediatric neuroradiologist.

### Clinical data

Patient clinical data sets were collected from online clinical records and stored in an anonymized database. Each patient performed every MRI during hospitalization, together with a complete clinical examination. For each MRI timepoint, we reviewed clinical data to infer disease-related disability as a global indicator of disease progression over time. Based on previous works [10], we defined patient disability as a variable with 3 grades (Table 1): autonomous unassisted ambulation (grade 0), autonomous ambulation with gait aid (grade 1), and absence of ambulation (patient on wheelchair) (grade 2). Furthermore, we collected epidemiological data from patient reports including age, disease duration, and genetic results. Disease onset was evaluated retrospectively based on KSS clinical diagnostic criteria, rather than genetic confirmation, because genetic testing was often delayed.

### Statistical analysis

A chi-square test was performed to investigate the prediction value of patient disability with brain score and spinal cord involvement. For those variables having a significant chi-square test, a contingency coefficient, phi factor, and Cramer’s *V* were also computed. We set the significance threshold (*p*) to 0.05. Statistical analysis was performed with SPSS Statistics (v.21, IBM, N.Y., USA).

## Results

Between January 2011 and March 2020, 18 patients were evaluated for the suspect of KSS at our institution. Seven patients were excluded because they lacked spinal cord evaluation. These subjects were consecutively acquired in the early recruitment period, when spinal imaging was not part of the standard protocol for KSS at our institution. Eleven patients met our inclusion criteria and were selected for the present study. The resulting population shared subjects with a recently published work [11]. The average patient age at the first MRI examination was 15 years for subjects with spinal cord involvement (4 females, 2 males) and 17 years for patients without spinal disease (2 females, 3 males). Disease duration at the first MRI was similar for subjects with spinal cord involvement (average 7.5 years) and without spinal disease (average 6.5 years). All patients underwent genetic testing for mitochondrial mutations and showed DNA deletions of variable base-pair length (Table 1). A definite diagnosis of KSS syndrome was reached in all patients by concomitance of clinical diagnostic criteria [1, 2] and genetic confirmation.

**Fig. 1:**
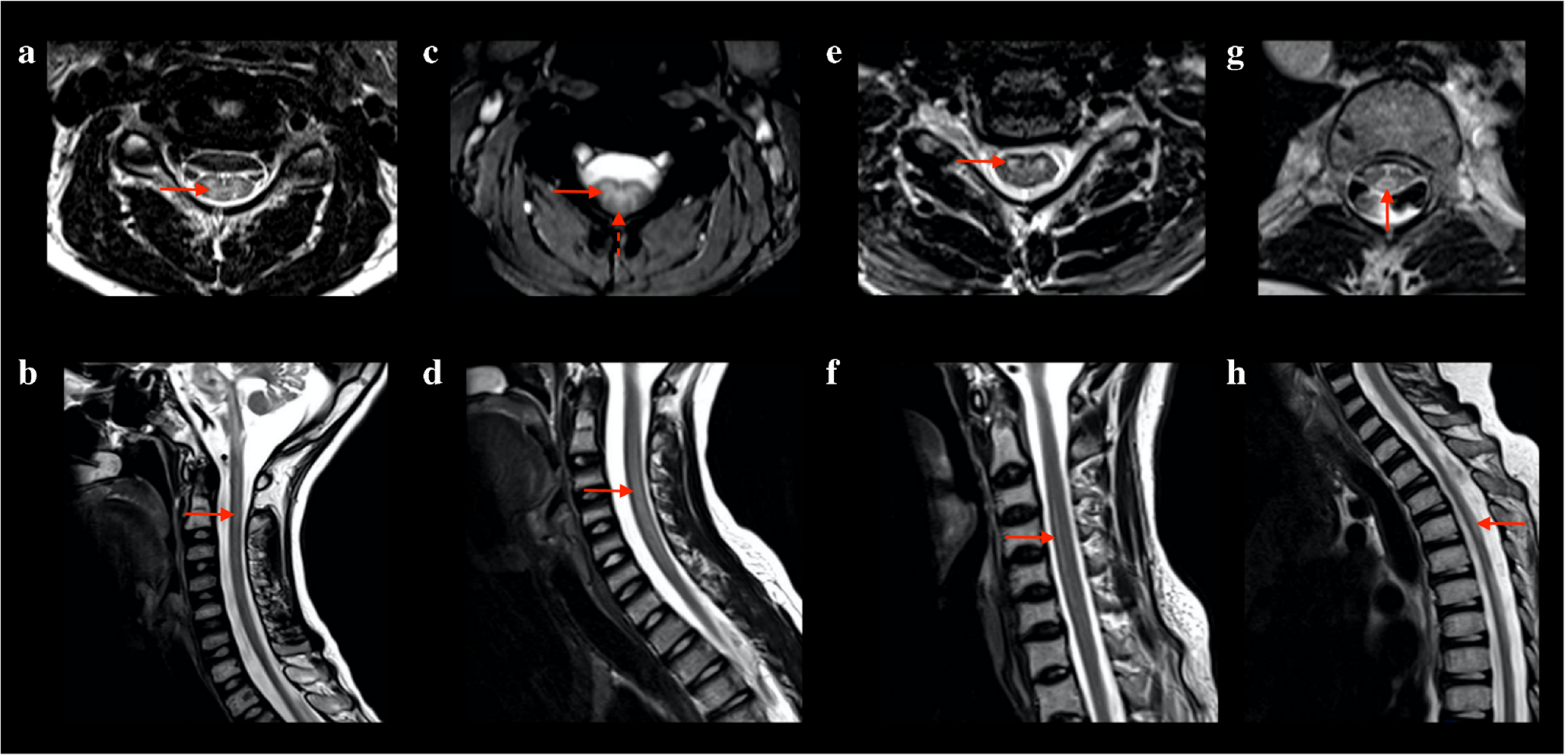
Axial and sagittal T2-weighted images displaying spinal cord disease in patients with KSS. Four predominant patterns were noticed: “H” pattern (solid arrows in image a and b); “H” plus pattern (image c and d, solid arrow in c pointing to gray matter and dotted arrow in c pointing to posterior columns); anterior pattern (solid arrows in e and f); posterior pattern (solid arrows in g and h)

**Fig. 2:**
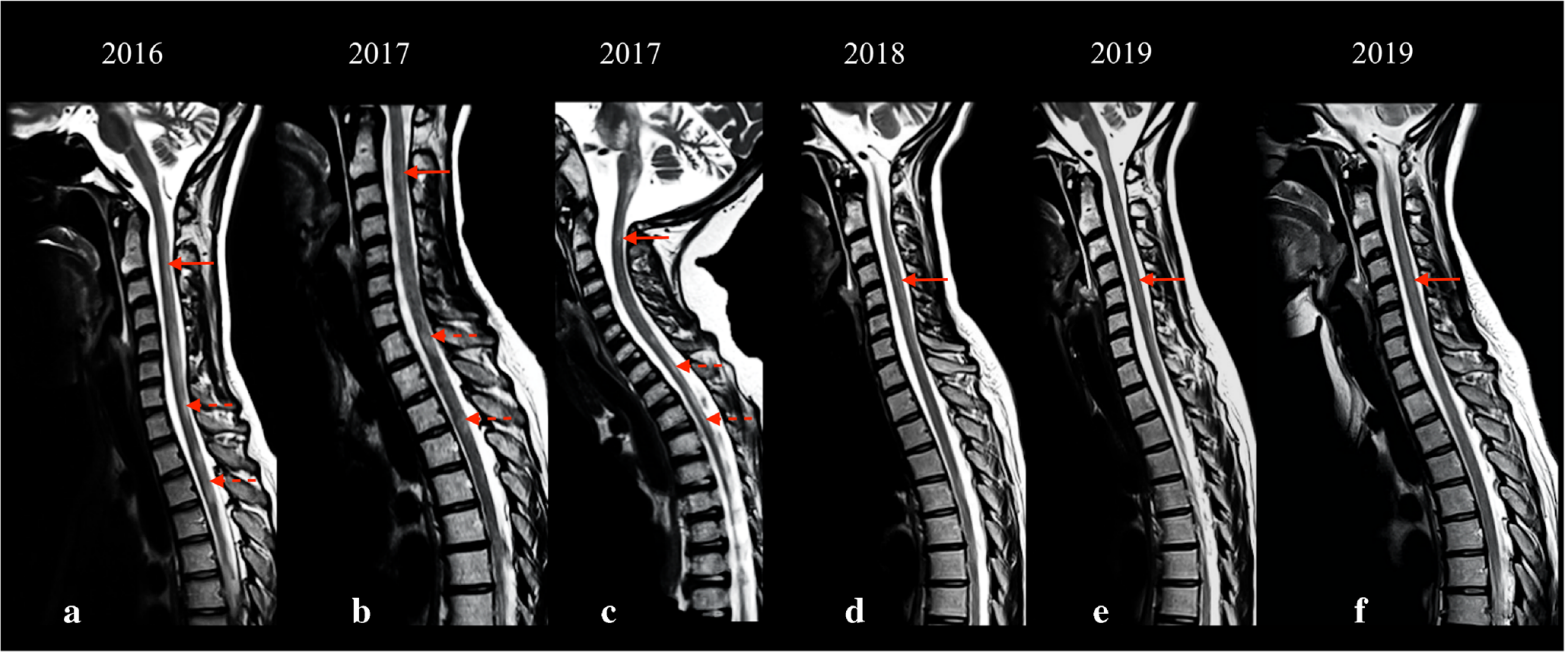
Sagittal T2-weighted images displaying spinal cord involvement across 4 years in a patient who had sudden onset of ambulation loss, with widespread “patchy” lesions in the cervical (images a, b, solid arrow) and dorsal (images a, b, dotted arrows) segments of the spinal cord. The lesions showed partial remission with more focal appearance (image c), and gradual worsening over the follow-up, with diffuse spinal cord hyperintensity (d–f, solid arrows)

**Fig. 3:**
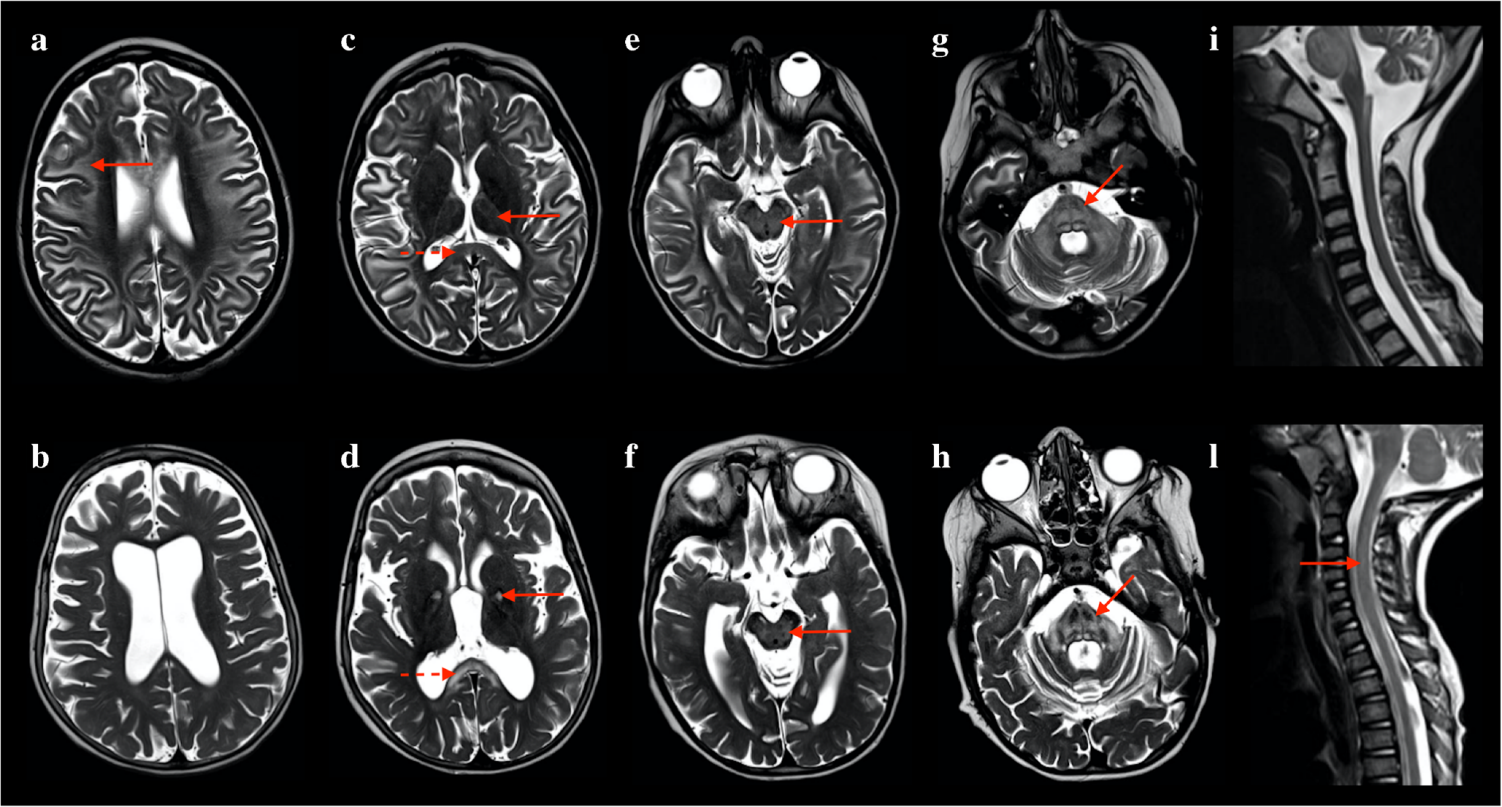
T2-weighted axial and sagittal images of two patients with KSS: the patient above (brain score = 12 points) showed widespread brain involvement, including subcortical white matter (a, solid arrow), thalami (c, solid arrow), and splenium of the corpus callosum (c, dotted arrow), midbrain (e, solid arrow), pons (g, solid arrow), no spinal cord lesions (i). The patient below (brain score = 8) showed slightly less brain involvement, including basal ganglia (d, solid arrow) splenium of the corpus callosum (d, dotted arrow), midbrain (f, solid arrow), pons (h, solid arrow), and spinal cord disease (l, solid arrow)

Six over eleven patients showed spinal cord lesions. Two patients showed grade 1 involvement (limited to the cervical segment) of the spinal cord, while four patients showed grade 2 involvement. Lesions were characterized by a hyperintense signal on T2-weighted images, with “patchy” or diffuse appearance. Four predominant patterns were noticed on axial T2-weighted images of the spinal cord (Fig. 1): exclusive involvement of gray matter (“H” pattern, 2 patients), involvement of gray matter and white matter of the posterior columns (“H” plus pattern, 2 patients), exclusive involvement of the posterior columns (posterior pattern, 1 patient), and exclusive involvement of the anterior columns (anterior pattern, 1 patient). Seven patients had multiple MRI examinations. Among patients with spinal cord lesions, 5/6 had minimum 1 and maximum 6 MRI follow-ups, with an average time interval of 14 months. Among patients without spinal cord lesions, 2/5 had an MRI follow-up, with an average time interval of 25 months. Qualitative longitudinal MRI evaluation of spinal cord lesions in patients with multiple scans showed spinal disease stability in 3 patients and disease progression in 2 patients. Disease progression was characterized by extension to other spinal segments. One patient displayed a peculiar behavior over time: sudden onset with widespread “patchy” lesions, apparent partial remission with more focal lesions, and gradual worsening over the last 2 years of follow-up, with widespread subtle T2 hyperintensity (Fig. 2). Despite the modifications in lesion appearance, the extension of spinal cord involvement in this patient was comparable over time. All 11 patients showed brain involvement, with similar scores for patients with and without spinal disease (average score of 9 points for patients with spinal cord involvement and 10 for patients without spinal lesions). From a qualitative evaluation, spinal cord disease seemed independent from the severity of brain involvement (Fig. 3). Different from spinal disease, brain involvement showed progression over time.

Four over six patients with spinal cord lesions showed disability, while autonomous ambulation was preserved in all patients without spinal disease. Two subjects with positive spinal cord showed grade 2 disability (need for a wheelchair): one of them showed disease progression over time, with an increment from grade 1 to grade 2 disability at the last follow-up. The other patient had an acute onset of complete ambulation loss (grade 2), initial partial recovery, and subsequent slow worsening, with grade 1 disability at the last follow-up (Fig. 2). In the remaining patients, the disability was mostly stable over time, reflecting the behavior of spinal cord involvement.

Data from each timepoint was considered for the statistical analysis (Table 1). A significant correlation was found between disability score and spinal cord involvement (*χ*^2^ = 7.64; *p* = 0.022) or brain score (*χ*^2^ = 26.85; *p* = 0.043). In addition, the values of strength of dependency were significant for both spinal (phi factor = 0.553, Cramer’s *V* = 0.553, contingency effect = 0.484; *p* = 0.022) and brain involvement (phi factor = 1.058, Cramer’s *V* = 0.748, contingency effect = 0.727; *p* = 0.043). Disability score dependence on brain score was also performed with the spinal cord as a cofactor to assess whether spinal disease influenced the correlation between brain involvement and disability. The results showed increased significance for brain score-disability correlation with the spinal cord as a cofactor (*χ*^2^ = 24.51; *p* = 0.017, phi factor = 1.201, Cramer’s *V* = 0.849, contingency effect = 0.767; *p* = 0.017).

## Discussion

Our results show that spinal cord involvement can be frequently observed in KSS and easily depicted by spinal MRI. From this retrospective analysis, spinal involvement seems to be independent from brain disease severity (Fig. 3); it may show different radiological patterns, involving white and gray matter structures (Fig. 1), and it may be correlated with patient disability. These results support the inclusion of spinal acquisitions to routine MRI in patients with KSS, including axial and sagittal T2-weighted images of the full spinal cord.

KSS spinal involvement is underreported in the literature, although it is well-known that clinical manifestations of the disorder may include sensory neuropathy, such as limb paresthesia and dorsal column loss [3, 12]. Moreover, several autopsy-based studies provide pathologic evidence for spinal involvement, characterized by white and gray matter vacuolization in the cervical segment, predominantly involving the posterior columns [5, 13–19]. Our findings in vivo support these observations. Compared with previous reports [6], in the population included in the study, spinal cord involvement was not limited to the cervical segment but often (4 cases) involved the thoracic and lumbar segment with a more extended pattern of injury. On the axial plane, we identified four radiological patterns of spinal disease: exclusive gray matter (“H” pattern), gray matter and white matter of the posterior columns (“H” plus pattern), exclusive posterior columns (posterior pattern), or anterior columns (anterior pattern) (Fig. 1). Moreover, multiple lesions may coexist in the same patient, showing more than one pattern.

These findings support the potential involvement of the spinal cord in mitochondrial disorders, such as LBSL (leukoencephalopathy with the brainstem, spinal cord involvement, and lactate elevation) syndrome due to DARS2 mutation where the posterior white matter columns are predominately involved [4, 20, 21]. However, as observed in our patients with KSS, the disease may also extend to other regions [6, 22]. Pathologic analysis of demyelinated areas from spinal nerve roots in KSS described the presence of glial bundles [16], an unspecific finding shared by multiple conditions, including degenerative and inflammatory disorders [23, 24]. Brockington et al. described capillary hyperplasia, infiltrate of macrophages, and reactive astrocytes in KSS spinal cord lesions, which may resemble inflammatory alterations [13]. However, no clear radiologic-pathologic correlation studies have been carried out so far in order to further characterize the spinal involvement in this rare disorder.

Statistical analysis showed significant correlations for spinal cord involvement and brain score with disability. Also, brain score correlation with disability was higher with spinal disease included as a co-factor, thus showing that spinal lesions may significantly contribute to the clinical deficit. While some authors reported that KSS clinical manifestations may not correlate with brain MRI changes [25], other studies achieved opposite results [26]. The discrepancy may partially depend on the fact that these evaluations were limited to the brain. Although the small sample of patients included in our study prevents us from drawing definite conclusions, our results may suggest that spinal disease has an impact on patient outcome, influencing disability, intended as loss of autonomous ambulation. Most of our patients showed a loss of autonomy over the years; however, an acute onset of disability was also observed in one patient, with a similar behavior compared with acute inflammatory disorders. This observation, together with previously reported pathologic data, further supports the hypothesis of an inflammatory response as a possible pathogenic cofactor for the development of spinal lesions in KSS and other mitochondrial diseases with spinal involvement (i.e., Leigh’ syndrome and Leber’s hereditary optic neuropathy) [13, 27–30].

Our study has some limitations. Due to the rarity of the disease, the amount of patients included in the study is small. This fact prevents us from drawing definite conclusions about our statistical correlations, requiring confirmation by future studies. Moreover, seven patients were excluded due to a lack of spinal imaging. These subjects were consecutively acquired in the early recruitment period, when spinal imaging was not part of the standard protocol for KSS at our institution. While we cannot clarify the presence of spinal lesions in the first seven patients, we can exclude that spinal imaging in the study population was performed based on clinical suspicion only. Although our patients performed multiple neurological examinations, a correlation between specific neurologic symptoms and spinal cord involvement was not performed in the study. This choice was due to the impossibility of differentiating between spinal and cerebral components of the symptoms. Electrophysiological data could help to discriminate between the two variables, but such data were not available in the majority of our patients due to the retrospective nature of the study. Future investigations may develop our results by means of a prospective analysis including extensive and standardized clinical evaluation, brain and spine imaging, and neurography.

## Conclusions

Spinal cord involvement is common in KSS and may show different patterns, including white and gray matter structures, with possible clinical correlates on patient disability. Therefore, we recommend including dedicated spinal cord sequences in routine MRI exams for patients affected by KSS. A comprehensive protocol should include sagittal and axial T2-weighted sequences from the cranio-cervical junction to the conus medullaris. Particularly, axial T2-weighted images should always be included in the MR protocol to allow optimal depiction of spinal lesions patterns. DWI sequences could add interesting data to further explore spinal cord involvement in KSS.
